# Safety and efficacy of a lattice-tip catheter for ventricular arrhythmia ablation: the AFFERA Ventricular Arrhythmia Ablation Registry (AVAAR)

**DOI:** 10.1093/europace/euaf139

**Published:** 2025-07-02

**Authors:** Frédéric Sacher, Andrea Sarkozy, Helmut Pürerfellner, Alexandra Steyer, Jonathan Lyne, Charlène Coquard, Romain Tixier, Nicolas Combes, Michel Haissaguerre, Michalis Efremidis, Josselin Duchateau, Victor Castro Urda, Claire A Martin, Marc Lemoine, Alejandro Carta Bergaz, Frank Bogun, Nick Linton, Nicolas Derval, Filip Schlosser, Thomas Pambrun, Jan Petru, Meleze Hocini, Luigi Pannone, Martin Mudroch, Josef Kautzner, Pierre Jais, Tobias Reichlin, Petr Neužil, Petr Peichl

**Affiliations:** Cardiac Arrhythmia Department, IHU Liryc, Univ. Bordeaux, INSERM 1045, CHU de Bordeaux, CMARY, ERN Guard Heart, REFER-RYTHMO Network, F33600 Bordeaux, France; Heart Rhythm Management Centre, Postgraduate Program in Cardiac Electrophysiology and Pacing, Universitair Ziekenhuis Brussel—Vrije Universiteit Brussel, European Reference Networks Guard-Heart, Brussels, Belgium; Department of Cardiology, Ordensklinikum Linz Elisabethinen, Linz, Austria; Cardioangiologische Centrum Bethanien (CCB), Frankfurt, Germany; Department of Pacing and Electrophysiology, Beacon Hospital, Dublin, Ireland; Cardiovascular Institute Paris-Sud (ICPS), Jacques Cartier Private Hospital, Massy, France; Cardiac Arrhythmia Department, IHU Liryc, Univ. Bordeaux, INSERM 1045, CHU de Bordeaux, CMARY, ERN Guard Heart, REFER-RYTHMO Network, F33600 Bordeaux, France; General and Interventional Cardiology—Rhythmology, Clinic Pasteur, Toulouse, France; Cardiac Arrhythmia Department, IHU Liryc, Univ. Bordeaux, INSERM 1045, CHU de Bordeaux, CMARY, ERN Guard Heart, REFER-RYTHMO Network, F33600 Bordeaux, France; Department of Electrophysiology and Pacing, Onassis Cardiac Surgery Center, Athens, Greece; Cardiac Arrhythmia Department, IHU Liryc, Univ. Bordeaux, INSERM 1045, CHU de Bordeaux, CMARY, ERN Guard Heart, REFER-RYTHMO Network, F33600 Bordeaux, France; Electrophysiology Unit, Cardiology Service, Hospital Puerta de Hierro Majadahonda, Madrid, Spain; Electrophysiology Department, Royal Papworth Hospital NHS Foundation Trust, Cambridge, UK; Department of Medicine, University of Cambridge, Cambridge, UK; Department of Cardiology, University Heart & Vascular Center Hamburg, University Medical Center Hamburg-Eppendorf, Hamburg, Germany; Servicio de Cardiología, Hospital General Universitario Gregorio Marañón, Centro de Investigación Biomédica en Red de Enfermedades Cardiovasculares, Madrid, Spain; Cardiac Arrhythmia Department, IHU Liryc, Univ. Bordeaux, INSERM 1045, CHU de Bordeaux, CMARY, ERN Guard Heart, REFER-RYTHMO Network, F33600 Bordeaux, France; Imperial College London, London, UK; Cardiac Arrhythmia Department, IHU Liryc, Univ. Bordeaux, INSERM 1045, CHU de Bordeaux, CMARY, ERN Guard Heart, REFER-RYTHMO Network, F33600 Bordeaux, France; Department of Arrhythmology/Cardiology, Institute for Clinical and Experimental Medicine (IKEM), Prague, Czech Republic; Cardiac Arrhythmia Department, IHU Liryc, Univ. Bordeaux, INSERM 1045, CHU de Bordeaux, CMARY, ERN Guard Heart, REFER-RYTHMO Network, F33600 Bordeaux, France; Department of Cardiology, Na Homolce Hospital, Prague, Czech Republic; Cardiac Arrhythmia Department, IHU Liryc, Univ. Bordeaux, INSERM 1045, CHU de Bordeaux, CMARY, ERN Guard Heart, REFER-RYTHMO Network, F33600 Bordeaux, France; Heart Rhythm Management Centre, Postgraduate Program in Cardiac Electrophysiology and Pacing, Universitair Ziekenhuis Brussel—Vrije Universiteit Brussel, European Reference Networks Guard-Heart, Brussels, Belgium; Department of Cardiology, Na Homolce Hospital, Prague, Czech Republic; Department of Arrhythmology/Cardiology, Institute for Clinical and Experimental Medicine (IKEM), Prague, Czech Republic; Cardiac Arrhythmia Department, IHU Liryc, Univ. Bordeaux, INSERM 1045, CHU de Bordeaux, CMARY, ERN Guard Heart, REFER-RYTHMO Network, F33600 Bordeaux, France; Department of Cardiac Electrophysiology, Inselspital, University of Bern, Bern, Switzerland; Department of Cardiology, Na Homolce Hospital, Prague, Czech Republic; Department of Arrhythmology/Cardiology, Institute for Clinical and Experimental Medicine (IKEM), Prague, Czech Republic

**Keywords:** VT ablation, Pulsed field ablation, Lattice-tip catheter, Safety

## Abstract

**Aims:**

The feasibility and safety of the lattice-tip catheter for ventricular arrhythmia (VA) ablation in humans remain largely unknown. This study aimed to assess feasibility, safety profile as well as patient outcomes after VA ablation with a lattice-tip catheter in a multicentre European registry.

**Methods and results:**

All 18 European centres using the AFFERA system in September 2024 agreed to participate. Clinical, procedural, and follow-up data (minimum 3 months) were systematically collected and analysed. A total of 126 patients (18% female; mean age 59 ± 16 years) underwent VA ablation using the lattice-tip catheter during the inclusion period. Ablation indications included ventricular tachycardia (VT) in 99, premature ventricular complexes (PVCs) in 23, and ventricular fibrillation (VF) in 4 patients. Major and minor acute complications were observed in 7 (6%) and 18 (14%) procedures, respectively. They included thrombo-embolic event (*n* = 2), major bleeding (*n* = 2), ventricular fibrillation induction (*n* = 1), tamponade due to epicardial access (*n* = 1), and cardiogenic shock due to prolonged VT mapping (*n* = 1). Within the first month post-procedure, three patients died [from multi-organ failure (*n* = 2) and sepsis (*n* = 1)], two had worsening heart failure, one myocardial infarction, one sepsis, and one major gastro-intestinal bleeding. After a mean follow-up of 5.6 ± 3.7 months, absence of recurrence was 78% for PVC, 70% for VT, and 100% for VF.

**Conclusion:**

In this complex population with refractory VA, ablation using the lattice-tip catheter appears feasible and relatively safe. In the absence of large, randomized trials, exhaustive registry is of key importance to ensure safety and efficacy of new catheter technologies.

## Graphical Abstract

**Graphical Abstract:**
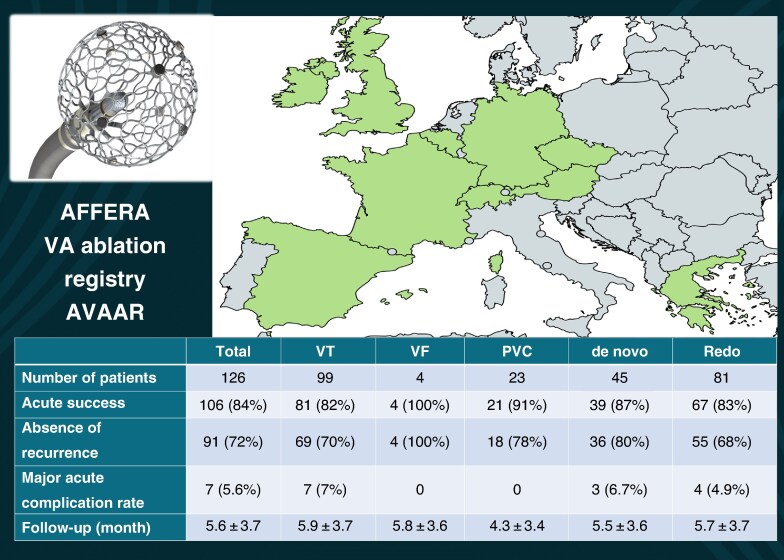
Summarizing the main findings of AVAAR.

What’s new?

- First multicentric institutional registry including all ventricular arrhythmia procedures using lattice-tip catheter [pulsed field ablation/radiofrequency (RF)].
- Safety in a very sick population is in the range of what has been reported with RF (6% of major complications).
- Short-term efficacy seems promising with an absence of ventricular arrhythmia (VA) recurrence in 36/45 (80%) patients for *de novo* VA ablation and 55/81 (68%) for redo procedures.

## Introduction

Catheter ablation is a cornerstone therapy for ventricular arrhythmias (VAs), yet with suboptimal outcomes, especially in patients with structural heart disease (SHD) and advanced cardiomyopathy. Recent advances include pulsed field ablation (PFA) with or without high power radiofrequency (RF) and novel catheter designs allowing for a larger effective surface area. The prototype of this technology is the lattice-tip catheter (Sphere-9, AFFERA, Medtronic) that promises improved procedural efficacy. This catheter is a component of the electro-anatomical mapping and ablation system AFFERA (Medtronic). However, real-world data assessing these new modalities, particularly in patients undergoing complex VA ablation, remain limited to small monocentric case series^1–8^ and systematic safety needs to be assessed.

The institutional European AFFERA Ventricular Arrhythmia Ablation Registry (AVAAR) was established to evaluate the safety of a lattice-tip catheter (Sphere-9, AFFERA, Medtronic) for VA ablation in a multicentre cohort across 18 European centres. This registry includes patients undergoing ablation for premature ventricular complexes (PVCs), ventricular tachycardia (VT), and ventricular fibrillation (VF).

In this study, we report outcomes from the AVAAR registry, focusing on procedural safety with early/late complications, and efficacy with VA recurrence during follow-up.

## Methods

### Population

All 18 centres equipped with the AFFERA system (Medtronic) by September 2024 were contacted to participate in a multicentre institutional registry on VA ablation: PVC, VT, and VF ablation. All centres accepted the invitation to participate. All patients in whom VA ablations were performed with this device until 31 December 2024 were included in the registry. Of note, two centres did not perform VA ablation during this period. The workflow of the VA ablation was left to the discretion of the operator. Demographic and procedural data were collected as well as any event occurring until March 2025 (minimum follow-up of 3 months).

### Catheter ablation procedure

**Figure 1:**
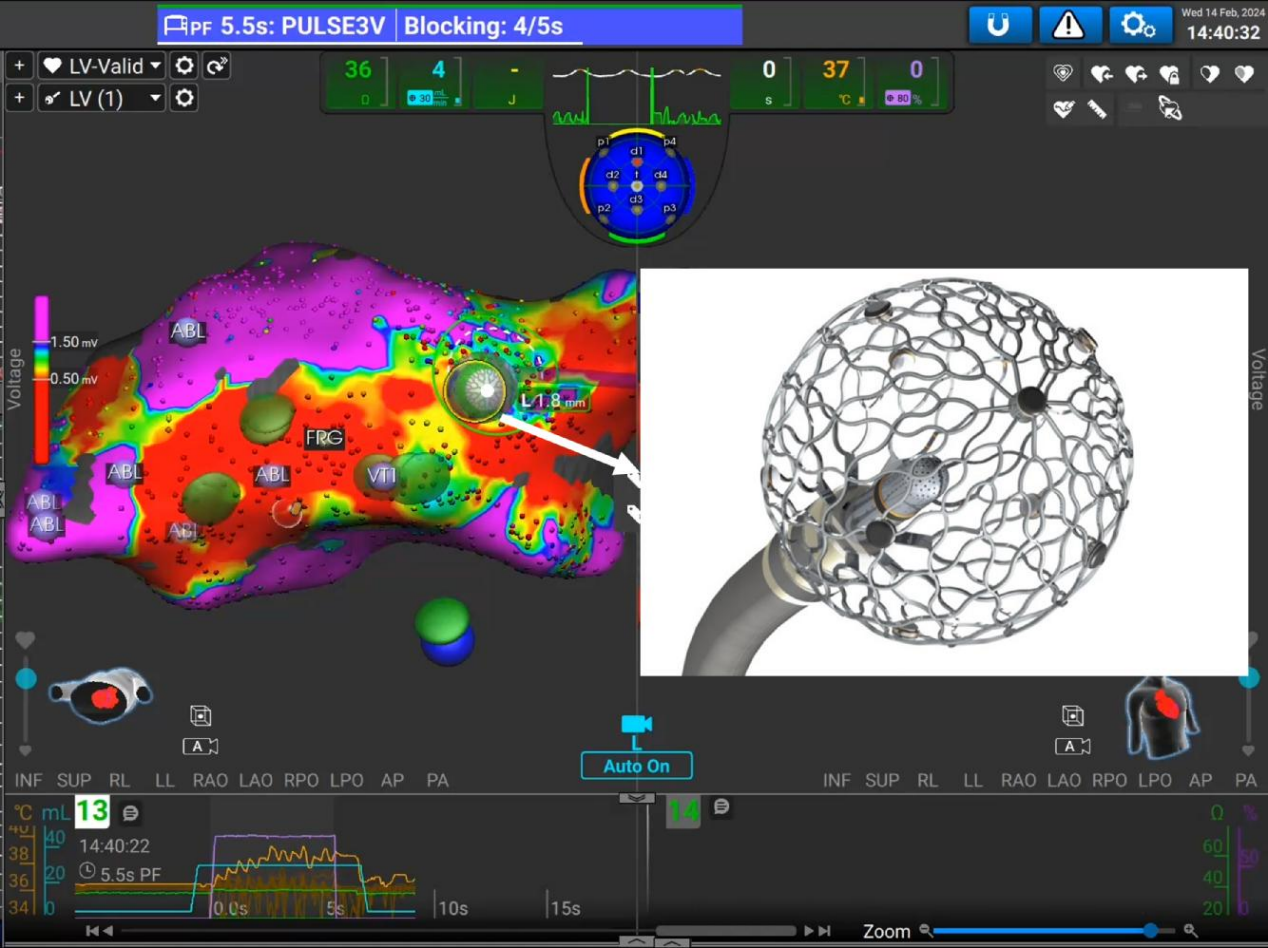
Electro-anatomical voltage map performed with the lattice-tip catheter with a picture highlighting the specific design of this catheter.

After informed consent was obtained, general anaesthesia or deep sedation was initiated. Following endocardial and/or epicardial access, a 9 mm lattice-tip catheter (Sphere-9, AFFERA, Medtronic) was used for mapping and ablation in association with a three-dimensional electro-anatomic mapping system (AFFERA, Medtronic). This system has been described earlier.^9^ Briefly, the lattice-tip catheter contains a magnetic sensor and nine microelectrodes on the surface of a collapsible frame that collect near-field unipolar electrograms against a central indifferent electrode (*Figure 1*). The electro-anatomical map is acquired rapidly by simultaneous recordings from all microelectrodes. At the same time, a dedicated algorithm automatically annotates local electrograms from each microelectrode.

At the beginning of the procedure, unfractionated heparin was administered as an initial bolus, and further doses were adjusted to maintain the activated clotting time (ACT) above 350 s. The exception was when accessing the epicardial space, which was performed prior to anticoagulation. Depending on the location of the arrhythmogenic substrate and/or the presence of peripheral vascular disease, the ablation catheter was deployed via an 8.5 French large sheath either transeptally and/or retrogradely to access the left ventricular endocardium. The epicardium was accessed through a transcutaneous subxiphoid access in case of epicardial substrate identified on imaging, suspected based on the underlying cardiomyopathy (CMP) or because of a previous failed procedure.

The use of imaging [intra-cardiac echocardiography (ICE), computed tomography (CT) or cardiac magnetic resonance imaging (CMR)], mapping, specific ablation strategies, ablation protocol in terms of the mode of energy applied (RF, pulsed field energy, or both), as well as application times and duration varied among the centres and was left to the discretion of the operator. However, PFA was not used in the vicinity of the conduction system.

### Clinical follow-up

Procedural data as well as events at discharge were collected. The patients were contacted at least 3 months after the ablation. The recurrence of VT/VF was assessed by clinical history, implanted cardioverter defibrillator (ICD) interrogation or via Holter monitoring for PVCs.

Complications were labelled as major if they led to death or to serious deterioration in the health of the patient (including life-threatening illness or injury, permanent impairment of a body structure or function, chronic disease or medical/surgical intervention to prevent injury, or permanent impairment of a body structure or function).

### Endpoints

The primary endpoint was the occurrence of complications (major and minor) during the procedure and up to 24 h after. Secondary endpoints included safety endpoints (procedure related complications occurring between 24 h and one month following the procedure, death, or cardiac transplantation during follow-up) and efficacy endpoints: (acute success defined as non-inducibility for VT ablation and PVC termination by energy application with absence of targeted PVC at the end of PVC/VF procedures) and absence of recurrence during follow-up (monitored via the ICD log or via ECG/Holter monitoring) without a blanking period.

Depending on the countries where the procedures were performed, non-opposition or consent of patients for data use was collected as recommended by national laws. European General Data Protection Regulation was followed.

### Statistical analysis

All variables were tested for normality with the Shapiro–Wilk test. Normally distributed variables were described as mean ± standard deviation and the groups were compared through paired or unpaired *t*-test as appropriate, while the non-normally distributed variables were described as median (inter-quartile range). Categorical variables were described as frequencies (percentages).

## Results

### Patient and procedural characteristics

**Figure 2:**
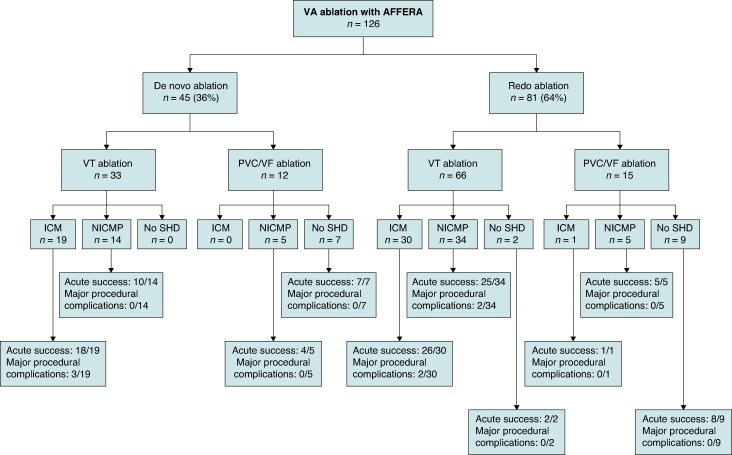
Flowchart of our population depending on *de novo* vs. redo procedure. VA, ventricular arrhythmia; VT, ventricular tachycardia; PVC, premature ventricular contraction; VF, ventricular fibrillation; ICM, ischaemic cardiomyopathy; NICM, non-ischaemic cardiomyopathy; SHD, structural heart disease.

The AVAAR registry included 126 patients (59 ± 16 years old, 23 females) with PVCs (*n* = 23), VTs (*n* = 99), or VF (*n* = 4) ablation. The underlying SHD was ischaemic CMP (40%), non-ischaemic CMP (NICMP) (46%), or absence of CMP (14%). Baseline characteristics are reported in *Table*  1. A total of 81 patients (64%) were referred after ≥1 previously failed VA ablation including 15% of the patients with >3 procedures (*Figure 2*). In these patients, the underlying substrate was for VT ablation: absence of scar (*n* = 2), ischaemic cardiomyopathy (ICM) (*n* = 30) with anterior (*n* = 9) inferior (*n* = 17), or lateral (*n* = 4) scars and for non-ischaemic cardiomyopathy (NICM) (*n* = 34): septal (*n* = 13), epicardial (*n* = 12), RV (*n* = 2), and peri-annular (*n* = 7) scars. Concerning patients with PVC/VF ablations, the underlying substrate was: absence of scar (*n* = 9), ICM (*n* = 1) with lateral scar and NICMP (*n* = 5) with papillary muscle (*n* = 2), septal (*n* = 1) or peri-annular (*n* = 2) scars. Of note, three patients referred for VT ablation had previous failed stereotactic body radiation therapy (SBRT). Procedural characteristics are reported in *Table 2*. Epicardial access was performed in 21 patients (17%) with the infusion of 40–50 cc of intra-pericardial saline depending on haemodynamic tolerance to help epicardial mapping in 19. The energy mode employed was RF energy alone (RF) (*n* = 19), PFA alone (*n* = 54), and both RF and PFA (*n* = 53). The mean total RF duration was 428 ± 354 s with 30–60 s per application in patients with RF delivery only. In patients with solely PFA, the mean number of PFA applications was 39 ± 30 with a maximum of 137 applications. In patients with both RF and PFA applications, the mean RF duration was 428 ± 282 s, and the mean number of PFA applications was 28 ± 30.

**Table 1:** Baseline characteristics of the population based on the indication

		VT (*n* = 99)	VF (*n* = 4)	PVC (*n* = 23)	Total (*n* = 126)
Population	Age (SD)	61 ± 16	38 ± 15	53 ± 16	59 ± 16
	Sex (female)	9 (9%)	3 (75%)	11 (48%)	23 (18%)
	Body mass index (kg/m^2^)	28.1 ± 4.3	23.3 ± 3.6	26.7 ± 5.7	27.7 ± 4.6
	Underlying substrate				
	None	2 (2%)	2 (50%)	14 (61%)	18 (14%)
	Ischaemic CMP	49 (49%)		1 (4%)	50 (40%)
	ARVC	5 (5%)			5 (4%)
	Inferolat subepicardial scar	3 (3%)		1 (4%)	4 (3%)
	Valvular disease	1 (1%)		3 (13%)	4 (3%)
	Congenital heart disease	4 (4%)			4 (3%)
	Hypertrophic CM	5 (5%)	1 (25%)		6 (5%)
	Idiopathic NICMP	30 (30%)	1 (25%)	4 (17%)	35 (28%)
	LVEF (%)	33 ± 12	66 ± 5	51 ± 15	37 ± 15
	Cl creat < 30 mL/min	8 (8%)	0	0	8 (6%)
	Previous embolic event (%)	14 (14%)	0	1 (4%)	15 (12%)
	Number of previous VA ablation procedure				
	None	33 (33%)	3 (75%)	9 (39%)	45 (36%)
	1	31 (31%)	1 (25%)	7 (30%)	39 (31%)
	2	19 (19%)		4 (17%)	23 (18%)
	>3	16 (16%)		3 (13%)	19 (15%)

SD, standard deviation; CMP, cardiomyopathy; ARVC, arrhythmogenic right ventricular cardiomyopathy; CL creat, clearance of creatinine; VA, ventricular arrhythmia.

**Table 2:** Procedural characteristics depending on indication

		VT (*n* = 99)	VF (*n* = 4)	PVC (*n* = 23)	Total (*n* = 126)
Procedure	General anaesthesia	96 (97%)	4 (100%)	21 (91%)	121 (96%)
	Type of access				
	RV only	8 (8%)	0	6 (26%)	14 (11%)
	Transeptal	67 (68%)	3 (75%)	12 (52%)	82 (65%)
	Retrograde aortic	27 (27%)	1 (25%)	7 (30%)	35 (28%)
	Epicardial	19 (19%)	1 (25%)	1 (4%)	21 (17%)
	Type of energy used				
	Only PFA	37 (37%)	4 (100%)	13 (57%)	54 (43%)
	Only RF	17 (17%)	0	2 (9%)	19 (15%)
	Both	45 (45%)	0	8 (35%)	53 (42%)
	Number of PFA application; (SD; min–max)	39 (32; 1–137)	23 (13; 9–40)	14 (11; 2–44)	33 (30; 1–137)
	RF duration (s) median (SD)	458 (307)	NA	230 (152)	428 (301)
	Number of RF application; (SD; min–max)	17 (12; 1–48)	NA	8 (6; 3–19)	16 (11; 1–48)
	Acute success				
	Yes	81 (82%)	4 (100%)	21 (91%)	106 (84%)
	No	11 (11%)	0	2 (9%)	13 (10%)
	Undetermined	7 (7%)	0	0	7 (6%)
	X-ray duration, min (SD)	11 (11)	19 (8)	10 (9)	12 (11)
	Procedure duration, min (SD)	174 (57)	210 (27)	128 (43)	167 (57)

PFA, pulsed field ablation; RF, radiofrequency; SD, standard deviation; NA, non-applicable.

Of note, electro-magnetic interferences occurred in the two patients with left ventricular assist device (Heart Mate 3). The catheter was only visible in the basal portions of the ventricle. Activation map was not feasible but some ablation tags were visible, though not projected on any anatomy.

### Complications

Major and minor acute complications are reported in *Table 3*. They were observed in 7 (6%) and 18 procedures (14%), respectively.

**Table 3:** Acute complications

Acute complications Procedure to Day 1	Procedure related	Potentially device related
Major complications 6%	Major bleeding (*n* = 2) Tamponade due to epicardial access with coronary laceration and myocardial infarction (*n* = 1) Cardiogenic shock (*n* = 1)	Thrombo-embolic event (*n* = 2) VF (*n* = 1)
Minor complications 14%	Femoral access (*n* = 3) Atelectasia (*n* = 1) Pericarditis (*n* = 1)	Transient ST elevation/depression post-application (*n* = 8) Biological haemolysis (*n* = 3) Mechanical LBBB (*n* = 1) LV chordae rupture by catheter manipulation but without consequence (*n* = 1)

Cerebral thrombo-embolic events occurred in two patients. Both patients had cerebellar strokes that resulted in full neurologic recovery. One 49-year-old male with a history of hypertrophic CMP and left ventricular aneurysm had a history of a left ventricular thrombus. No thrombus was identified on a contrast enhanced transthoracic echocardiogram immediately prior to ablation nor on an intra-cardiac echocardiogram during the ablation procedure. The procedure was performed with an International Normalised Ratio of 2.87 and an ACT > 350 s. Transeptal access was used to deliver 28 RF and 19 PFA applications. The procedure time was 133 min. The second patient was a 64-year-old male with ICM and a left ventricular (LV) ejection fraction of 30%. Pre-procedural imaging with CT did not show a ventricular thrombus. An ACT > 350 s was achieved before LV mapping via a transeptal access. Thirty-eight RF and nine PFA applications were delivered. The procedure lasted 210 min. After extubation, a right cerebellar deficit was noted that recovered during the hospital stay. The patient was discharged on Day 7. No clear explanation was identified in either of these two patients.

**Figure 3:**
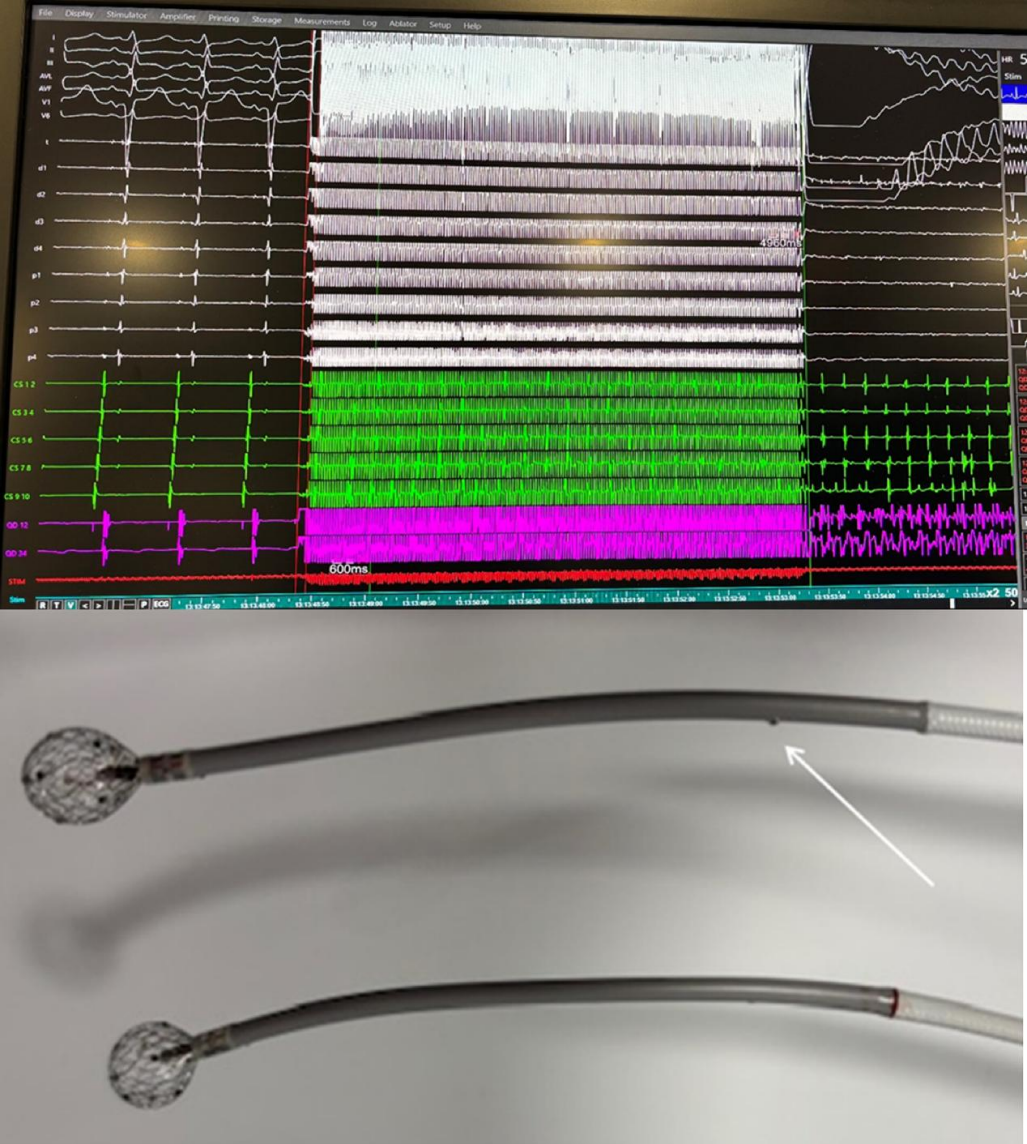
Tracings of a 71-year-old with induction of VF and AF during RF application due to current leakage due to damage of the shaft as shown by the white arrow. The catheter below is a standard one, without shaft damage.

Simultaneous VF and AF were induced during RF application in one patient due to current leakage. This was a long procedure with high catheter constraints first of inferior scar related multiple morphology VTs via transeptal approach, with switch to steerable sheath due to difficult reach of target regions. During RF application for easily inducible AVNRT in the slow pathway region, appearance of electrical noise and simultaneous induction of VF and AF was observed. Radiofrequency application and procedure were terminated and patient recovered uneventfully. At close visual inspection of the Sphere-9 catheter, a rupture of the shaft isolation was observed (*Figure 3*). The problem has been identified, production controlled, and close monitoring instituted. Since then, this problem has never arisen again.

Two acute major bleeding events were encountered and were due to femoral access one requiring blood transfusion, and the other additional vascular surgery.

An acute cardiogenic shock occurred in a patient (LVEF 23%) with prolonged VT mapping necessitating cardiopulmonary resuscitation and extracorporeal membrane oxygenation. This patient eventually recovered and was discharged.

Direct coronary artery injury occurred in one patient due to laceration during an epicardial access. It resulted in tamponade and myocardial infarction. In eight other patients, transient ST modification (elevation/depression) was noticed. In five patients, it happened after epicardial PFA application without spasm in one and with spasm identified in the four others. In two patients, ST changes happened after endocardial RV PFA applications. In one of these patients, PFA was applied in the RVOT and spasm of the ostium of the right coronary artery was subsequently noted. The other patient had PFA application on the moderator band but no spasm was identified and ST segment normalized spontaneously. The last patient had ST depression following endocardial LV PFA applications that resolved spontaneously without spasm identified. In all cases in which spasm was identified, it resolved after nitrates administration. In case of spasm, nitrates (1 mg) were administered and repeated till hypotension developed/spasm resoluted.

While the protocol did not require systemic assessment of haemolysis, it was reported in three patients including the patient with 137 PFA applications. There was no associated acute renal failure in any patient.

Another complication was the rupture of one of the false tendons. During manipulation in the LV (using a transeptal approach), resistance was felt during movement of the catheter in the base of the posteromedial papillary muscle. Subsequently, the catheter was pulled out from LV, but on ICE, rupture of one of the false tendons was noted. No change in mitral valve insufficiency was noted (remained 1–2/4), and LVEF remained 30–35% (one year follow-up echo data).

Delayed complications possibly related to the ablation procedure occurred in eight patients (6%) in the month following the procedure (*Table 4*).

**Table 4:** Major complications occurring between Days 1 and 30

Major complications from Day 1 to 1 month	Death (*n* = 3) Heart failure after Day 1 (*n* = 2) Myocardial infarction (*n* = 1) Sepsis (*n* = 1) Major gastro-intestinal bleeding (*n* = 1)

Three patients died during the month following the procedure; first, the 75-year-old male with coronary artery laceration during epicardial access. He experienced myocardial infarction, pericardial effusion, and then cardiogenic shock complicated by sepsis and finally death; secondly, a 57-year-old male with ischaemic CMP (LVEF 15%) and HF history. He received 76 PFA applications and 6 min of RF. He experienced a HF episode at post-operative Day 1 and finally died of multi-organ failure in intensive care following his HF episode treatment; and thirdly, another 71-year-old male with myelofibrosis and valvular CMP (LVEF 55%) who received 15 PFA applications and 4 min of RF finally died of sepsis with multi-organ failure and tumorlysis with leukostasis.

Decompensated HF occurred in two additional patients: a 75-year-old female with a history of HF and ischaemic CMP (LVEF 20%) who received a blood transfusion after bleeding from vascular access. This occurred 7 days post-ablation. In the other patient, a 71-year-old male with NICM (LVEF = 50%), HF occurred on post-operative Day 4, without prior history after he received 31 PFA applications and 13.5 min of RF during a 244 min procedure.

The three other complications that occurred in the month following the procedure were myocardial infarction, major gastro-intestinal bleeding, and sepsis. A patient with myocardial infarction had a history of coronary artery disease with coronary artery bypass graft. He had four previous RF ablations and a SBRT procedure. Two weeks after the PFA procedure, he had intermittent chest pain with troponin 800 ng/L (normal < 14 ng/L). He finally had coronary angiography and subsequently PCI of the venous graft to the marginal branch. This appeared unrelated to the ablation procedure.

Finally, one additional patient died 5 months following the ablation procedure from VT storm after two additional failed ablation procedures, and two patients had cardiac transplant (anticipated before ablation) during follow-up, 2 and 3 months post-ablation.

### Efficacy

Acute success was achieved in 81 (82%) patients referred for VT ablation, 4 (100%) for VF, and 21 (91%) for PVC ablation. During a minimal follow-up of 3 months, 69/99 (70%) of patients treated for VT ablation (mean follow-up 6 ± 4 months), 4/4 (100%) treated for VF (mean follow-up 6 ± 4 months), and 18/23 (78%) treated for PVC (mean follow-up 4 ± 3 months) remained free from their VA. Looking at efficacy depending on the number of previous procedures, absence of recurrence was achieved in 36/45 (80%) patients with *de novo* VA ablation vs. 55/81 (68%) in patients with redo procedures.

## Discussion

### Main findings

This institutional multicentre European registry provides a comprehensive evaluation of VA ablation procedures and patient outcomes using a lattice-tip catheter. Despite the presence of a challenging patient population characterized by advanced structural heart disease, high prevalence of redo procedures, and substantial comorbidities, an acute and short-term success rate of 84% and 72% could be achieved. Major peri-procedural complications occurred in 6% of patients. These findings suggest that use of the AFFERA ablation system in patients with VA is feasible with a reasonable safety profile.

### Safety profile and complications

While complications occurred, the prevalence of major complications is comparable to that reported in the ablation arms of several VT ablation studies using RF energy in a similar patient population (3.8–11.2%).^10–17^ A few case series^1–8^ using various PFA systems (catheter and generators) reported the feasibility to treat VA with PFA. Due to the heterogeneity of the different PFA systems, the efficacy/safety profile may not be uniform. Therefore, systematic assessment of each system is critical. The major strength of the present registry is the inclusion of all cases of VA ablation procedures performed in clinical practice worldwide until December 2024 with the same dual energy lattice-tip catheter (Sphere-9, AFFERA, Medtronic). This allowed a systematic evaluation of various operators’ experiences using a promising new technology, which is essential to optimize the workflow and avoid complications.

Safety outcome was the primary purpose of this registry, keeping in mind the challenging patient population with most patients having failed prior procedures In our series, cerebrovascular events occurred in two patients (1.6%). In the literature with standard RF catheters, it varies from 0–2.8%.^12–17^ Notably, both stroke cases reported here, involved significant RF and PFA lesion delivery, and both patients fully recovered neurologically. Careful patient selection, meticulous anticoagulation management, and pre-procedural imaging to assess for thrombi may further help to minimize thrombo-embolic events.^18^

Peri-procedural cardiac decompensation occurred in several patients. This is also a complication that is well known with established risk factors that may be independent on the ablation mode (prolonged VT mapping, fluid infusion, fragile population, general anaesthesia).^19,20^ General anaesthesia, which is almost always used for PFA procedures, may substantially increase the risk for peri-procedural HF.^21^ Additionally, with the development of PFA and its large electrical field, concern about the risk of cardiac stunning has emerged especially when treating large area VA ablation, scar border zone, or healthy tissue. It is certainly worth remaining vigilant about this risk especially in patients with low ejection fraction. If a large number of applications on the border zone are required, the use of RF energy might be a better strategy. In any case, we should always limit the number of applications in healthy tissue/border zone to a strict minimum.

Feasibility and safety of epicardial mapping and ablation with a lattice-tip catheter have not been extensively reported so far. From a pre-clinical study,^22^ we know that epicardial PFA lesions are comparable to endocardial ones. However, there are no clinical data outside small case series. Epicardial access has been performed in 21/126 procedures in our study. Based on our experience and because of the shape of the catheter tip, intra-pericardial saline infusion (around 40–50 cc depending on haemodynamic tolerance) prior insertion of the lattice-tip catheter may help smooth manipulation and epicardial mapping with the lattice-tip catheter. Moreover, after ablating epicardially, particular attention should be paid to the catheter tip before returning endocardially as tissue was identified on it after epicardial ablation in one patient. Another concern is coronary artery spasm that has been described as a potential complication when delivering PFA endocardially in the atria facing coronary arteries. However, effect on ventricular myocardium is less clear.^23,24^ In our series, ST changes (elevation/depression) after energy application was seen in eight patients. Of note, in five patients, PFA was applied on the epicardium,^7^ however in the three other patients, PFA applications were only endocardial but with proven spasm in only one. The remaining question is the potential effect of PFA on coronary arteries on the longer term. Kawamura *et al*.^25^ showed in swine with another PFA system that coronary artery spasm can be seen in up to 33% with PFA applied from the ventricular endocardium. Interestingly, at one month, PFA applications resulted in minimal to mild histological coronary artery changes (focal medial fibrosis and intimal hyperplasia) in 18% of applications. However, they did not identify any change with another PFA catheter. These histological findings were also present in 42% of RF applications. Therefore, it is difficult to make any conclusions about any mid- to long-term effects of PFA application on the coronary arteries so far^26^ and whether direct applications from the epicardium could have a different impact.

Importantly, no case of haemolysis with clinical consequences were observed, a finding of significance given prior reports related to PFA energy^27,28^ and myocardial tissue interactions. This supports the notion that the AFFERA system’s energy delivery parameters are generally safe, even in procedures with multiple PFA applications. There are now more data showing that haemolysis can vary with PFA depending on the system (catheter and PFA recipe).^29,30^

Catheter entrapment as described with other catheters in 0–0.7% of ablation procedure,^12,31^ is certainly possible with this lattice-tip catheter. In our registry, the catheter was trapped in one patient but it was possible to remove it, at the cost of a rupture of a false tendon, fortunately without clinical consequences. To avoid this complication, it is critical not to continue to turn the catheter in the same direction especially when it is curved and close to the mitral apparatus.

Mortality during the early follow-up period was observed in three patients, with an additional one at 5 months because of electrical storm despite redo ablation. Additionally, two patients underwent cardiac transplantation. Deaths were multifactorial, including complications of sepsis, myocardial infarction secondary to epicardial access injury, and progressive heart failure. Their occurrence reflects not only procedural risks but also the advanced baseline severity of cardiac disease in the population enrolled. While mortality is concerning, it is consistent with historical reports for high-risk VT ablation cohorts, where in-hospital mortality can range from 0–3%, and one-year mortality 3%–35%^10,12–16^ depending on the population included.

### Efficacy

**Figure 4:**
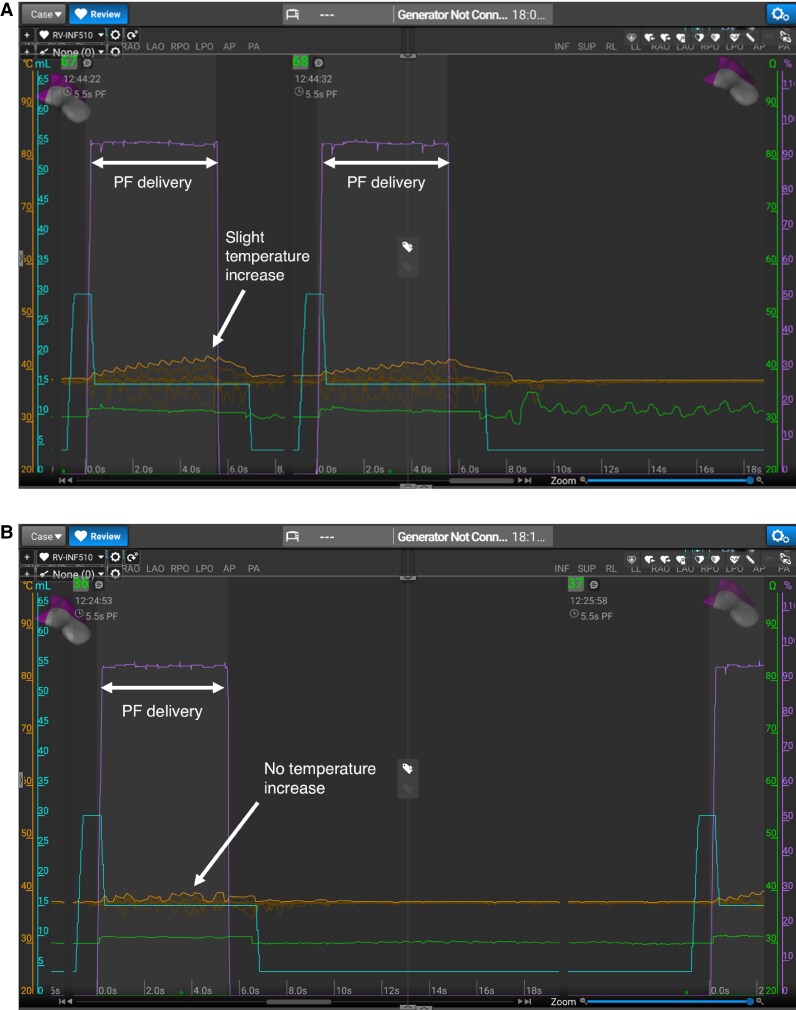
Different profile of PFA applications with the lattice-tip catheter. Panel *A* shows a slight temperature increased (orange tracings) typical of effective application whereas panel *B* is typical of suboptimal application in the absence of temperature change (orange tracings).

Procedural efficacy was notable, with 72% of patients free from VA recurrence at 5.6 ± 3.7 months. This is particularly meaningful given the high proportion of patients with previous failed ablation and with complex substrate. Apart from the type of energy used, the design of the catheter is very well suited for VA ablation.^32^ Despite learning a lot from pre-clinical studies, the optimal recipe to obtain safe transmural and durable ventricular lesions in a clinical setting needs to be refined. One concern is the possible transient effect of PFA, if application is not optimal. In some patients, areas recovered after PFA application as identified during remapping at the end of the procedure (>2 h after application) or during a redo procedure in case of recurrence. Retrospectively, contact may not have been optimal on these sites as assessed by the absence of temperature increase during application (*Figure 4*). Contact is important for effective and durable lesions even with PFA.^33^ The impact of force is however less clear. In healthy swine atrium with a focal dual energy catheter but without repetition, lesion depth, width, and volume were larger with higher degrees of contact force.^34^ However, an important parameter of lesion size with PFA is repetition. When using PFA in the ventricle, four repetitions seem to create the deepest lesion^22,35,36^ in healthy swine ventricular myocardium. Acute lesions showed a well-demarcated necrotic core without coagulation necrosis while chronic lesions showed tissue thinning with fibro-fatty replacement. Moreover, superficial scar or previous ablation does not impair lesion depth obtained by PFA applications.^22,37^ In scar tissue and compared to RF, PFA produced uniform and well-demarcated lesions exhibiting irreversible injury with deeper and more transmural lesions.^38,39^ An important observation from this registry is the mixed use of RF and PFA applications within the same procedure. While the flexibility to combine energy modalities may allow for tailored lesion sets targeting both superficial and deep arrhythmic substrates, it also complicates the interpretation of the specific contribution of PFA vs. RF to procedural success or failure. A pre-clinical study^40^ investigated the effect of sequential, colocalized RF and PFA on cardiac lesion size and histology in swine. They found that combining RF and PF—regardless of the sequence—increased lesion depth and width significantly compared to either modality alone, with histological analysis showing central thermal necrosis (typical of RF) surrounded by zones of PFA-induced damage.

Finally, to increase efficacy and limit the risk of VA ablation, several strategies have been developed over the years concerning mapping and ablation.^41^ Mapping evolution allowed to better identify target with the use of high density mapping and imaging.^18,42^ Concerning ablation different energies have been developed to create deeper/larger lesion such as ethanol infusion,^43–45^ stereotaxic radiation therapy,^46^ ultralow cryo-energy,^47,48^ and now PFA. However, it should be reinforced that procedural success and patient outcomes do not only depend on lesion depth by employing a particular energy source or catheter technology but also on operator experience, mapping strategies, patient selection, timing of intervention, and adjunctive management of heart failure and comorbidities. The AVAAR registry demonstrates that even with advanced technology, the inherent challenges of treating a fragile patient population persist, emphasizing the need for continued innovation across all facets of care.

### Limitations

Although the registry involved several highly experienced centres, the study was not randomized, and there was no control group. Because we wanted to report all cases of VA ablation performed with this new system, we included all centres using this tool as of September 2024 that may have introduced a centre-level bias. The number of patients, while larger than any series, remains modest for robust subgroup analyses and would require randomized control trial to identify differences compared to usual RF tools. Procedural protocols were not standardized across the centres, potentially introducing heterogeneity in technique and outcome. Furthermore, follow-up was relatively short, and longer-term data are needed to fully assess the durability of arrhythmia suppression and the long-term safety profile of the AFFERA system.

## Conclusions

In this complex population with refractory VA, ablation using a lattice-tip catheter appears feasible with a reasonable safety profile. In the absence of large randomized trials, assessment of procedural data and outcomes in the form of an exhaustive registry is of key importance to assure safety and efficacy of new catheter technologies.

## Data Availability

The data underlying this article were collected from the participating centres. Data will be shared on reasonable request to the corresponding author after agreement of the participating centres.
